# Reduced CSF turnover and decreased ventricular Aβ42 levels are related

**DOI:** 10.1186/1471-2202-12-42

**Published:** 2011-05-13

**Authors:** Jean-Marie Serot, Johann Peltier, Antony Fichten, Nelly Ledeme, Anne-Marie Bourgeois, Pierre Jouanny, Patrick Toussaint, Daniel Legars, Olivier Godefroy, Jean-Claude Mazière

**Affiliations:** 1 Department of Gerontology, CHU Amiens, France; 2 Department of Neurosurgery, CHU Amiens, France; 3 Department of Biochemistry, INSERM ERI-12, CHU Amiens, France; 4 Department of Neurology, CHU Amiens, France

## Abstract

**Background:**

The appearance of Aβ42 peptide deposits is admitted to be a key event in the pathogenesis of Alzheimer's disease, although amyloid deposits also occur in aged non-demented subjects. Aβ42 is a degradation product of the amyloid protein precursor (APP). It can be catabolized by several enzymes, reabsorbed by capillaries or cleared into cerebrospinal fluid (CSF). The possible involvement of a decrease in CSF turnover in A4β2 deposit formation is up to now poorly known. We therefore investigated a possible relationship between a reduced CSF turnover and the CSF levels of the A4β2 peptide.

To this aim, CSF of 31 patients with decreased CSF turnover were studied. These patients presented chronic hydrocephalus communicating or obstructive, which required surgery (ventriculostomy or ventriculo-peritoneal shunt). Nine subjects had idiopathic normal pressure hydrocephalus (iNPH), and the other 22 chronic hydrocephalus from other origins (oCH).

The Aβ42 peptide concentration was measured by an ELISA test in 31 ventricular CSF samples and in 5 lumbar CSF samples from patients with communicating hydrocephalus.

**Results:**

The 5 patients with lumbar CSF analysis had similar levels of lumbar and ventricular Aβ42. A significant reduction in Aβ42 ventricular levels was observed in 24 / 31 patients with hydrocephalus. The values were lower than 300 pg/ml in 5 out of 9 subjects with iNPH, and in 15 out of 22 subjects with oCH.

**Conclusion:**

The decrease of CSF Aβ42 seems to occur independently of the surgical hydrocephalus aetiology. This suggests that a CSF reduced turnover may play an important role in the decrease of CSF Aβ42 concentration.

## Background

Alzheimer's disease (AD) is characterized by the presence of diffuse and senile dense-core plaques. Senile plaques may also be seen in non-demented elderly patients. According to the « amyloid theory », the appearance of Aβ42 deposits is considered as the crucial event in the evolution of AD [1].

Aβ42 can be reabsorbed by capillaries via specific receptors, the most charaterized being LRP-1 [2], degraded by several enzymatic pathways or cleared into cerebrospinal fluid (CSF) [3]. In AD patients, Aβ42 production is similar to that of cognitively normal controls, but its clearance is impaired [4]. The CSF turnover is defined by the volume of CSF produced in 24 hours divided by the volume of the CSF space. It can be estimated to 4 volumes per day for healthy young adults [5]. The consequences of decreased CSF turnover on the clearance of Aβ42 were still unknown.

In vitro, Aβ42 monomers, incubated into CSF, build spontaneously oligomers and then fibrils; these phenomena are time-dependant [6,7]. Experimentally, it has been shown that brain amyloid deposits accumulate in rats with kaolin-induced hydrocephalus [2]. In this model, immunochemistry shows an accumulation of Aβ42 without significant increase in APP expression. This is associated to an increase in p-tau and a loss of dendritic staining. Altered CSF turnover seems to affect Aβ42 clearance and to favour amyloid cerebral deposit formation [8].

Several human types of chronic hydrocephalus with altered CSF turnover are known, such as idiopathic Normal Pressure Hydrocephalus (iNPH), and those following meningeal haemorraghe or related to surgery for Sylvius acqueduct stenosis, or brain tumors. iNPH is a rare disease (about 1 case for 200000 people), described in 1965, and characterized by a clinical triad of mental alteration, gait disturbance and sphincter dysfunction [9]. In this affection, ventricular dilatation has also been described, with a normal pressure of lumbar CSF, a perturbation of CSF resorption, and a correlative reduction in CSF turnover to less than 1.5 volume per day instead of 4 volumes as seen in young controls [5]. In AD, CSF turnover is also reduced and can be estimated to less than 1.5 volume per day [5,10].

In order to investigate a possible involvement of a decrease in CSF turn-over on Aβ42 clearance, we studied the ventricular levels of this peptide in patients suffering from chronic hydrocephalus, which required surgery (ventriculostomy or ventriculo-peritoneal shunt). We compared lumbar and ventricular CSF levels in some patients with communicating hydrocephalus, we found ventricular levels are decreased in iNPH and similar to those measured in patients suffering from chronic hydrocephalus, communicating or obstructive.

## Methods

The prospective study was performed on a panel of 31 consecutive patients (men: 17, women: 14), aged from 28 to 86 years (mean age 64.9 ± 15.5 years; median 68 years). All of them were affected with chronic hydrocephalus from various aetiologies, which required either a ventriculostomy or a ventriculo-peritoneal shunt. In Phase Contrast MRI, all the patients had ventricular dilatation and periventricular edema due to transependymal resorption of CSF; the patients with communicating hydrocephalus were also characterized by hyperdynamic aqueductal CSF flow [11].

The experimental protocol was the following: during the surgery, 3-4 cm^3 ^of CSF were aspired, as usual, in order to check the position or the functionality of the shunt. After sampling for cyto-bacteriological analysis, residual CSF was immediately aliquoted in polypropylene tubes placed on ice, and transported to the Biochemistry Laboratory, where it was centrifuged at 4000 × g for 10 min., then stored at - 80°C. This study was achieved according to the French bioethic rules for clinical studies on tailing samples. All the patients gave first their agreement for this protocol.

The respective diagnoses for these patients were: 9 iNPH, and 22 chronic hydrocephalus from other origins (oCH): 3 related to a meningitis or a meningeal haemorrhage, 4 due to stenosis of the Sylvius aqueduct, 10 from benign or malignant tumor origin, 2 following a meningioma surgery, 2 due to a Arnold-Chiari syndrome or a neonatal hydrocephaly and one secondary to the rupture of a dermoid cyst. Five out of these patients (3 with iNPH and 2 with hydrocephalus post subarachnoidal haemorraghe, all older than 65 years) also benefited of a prior subtractive lumbar tap.

The concentrations of Aβ42 were measured using a commercial ELISA test (Innotest β-amyloid [1-42] from Innogenetics, Ghent, Belgium). With this method, the intra-assay variability coefficient is less than 6%. The range for the titration zone is 125-2000 pg/ml. CSF lumbar Aβ42 levels higher than 500 pg/ml are considered as normal [12].

## Statistics

Ventricular Aβ42 values in the two groups (iNPH, oCH) were compared using the Student' *t *test corrected for unequal variance. Correlations between values of Aβ42 measured in lumbar and ventricular CSF, were analyzed using the Pearson's test. P < 0.05 were considered as significant.

## Results

**Figure 1:**
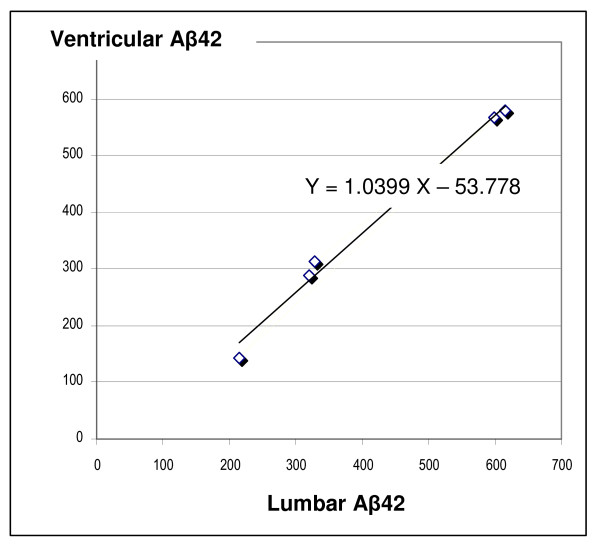
**Correlation between lumbar and ventricular CSF levels of Aβ42 (values are expressed in pg/ml; R^2 ^= 0.989, P < 0.0001)**.

**Figure 2:**
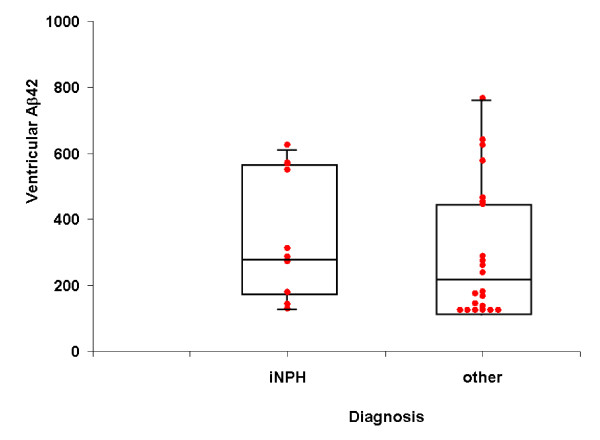
**Comparison of ventricular Aβ42 levels between iNPH and other hydrocephalus**. The space between 2 horizontal lines is the interquartile range. The boxes represent the 25^th^, 50^th ^and 75^th ^percentiles (values are expressed in pg/ml).

There was no correlation between Aβ42 levels and age. In the 5 patients with lumbar CSF analysis, lumbar and ventricular Aβ42 levels (respectively 415.6± 180.8 pg/ml and 378.4± 189 pg/ml; p>0.05) were found similar and highly correlated (r = 0.99; Figure 1). Based on this fact, Aβ42 ventricular values lower than 500 pg/ml were considered as reduced. This is the case for 24/31 patients (77%), with values under 300 pg/ml in 20 patients (64%) including 5 out of 9 iNPH (55%) and 15 out of 22 oCH (68%; figure 2).

It can also be observed in Figure 2 that no significant difference was found between the iNPH and oCH groups for ventricular Aβ42 levels (respectively 297.1 ± 343.3 pg/ml and 206.7 ± 194.8 pg/ml, p > 0.2; median values 286 and 211 pg/ml).

## Discussion

This work shows that ventricular and lumbar levels of Aβ42 are closely related in communicating hydrocephalus; they are low in iNPH, and comparable to those of other surgical hydrocephalus.

The main limit of this study is that, for ethic reasons, we cannot compare the obtained data to the concentrations existing in "normal" subjects. CSF is a transport medium for numerous molecules (nutriments, growing factors, terminal products of brain cell metabolism). The ventricular and lumbar protein concentrations are often different, depending upon their origin. Indeed, brain proteins are considered to generally exhibit a higher level in intraventricular CSF, whereas blood proteins are more concentrated in lumbar CSF [13,14]. However, there are many exceptions; for example, concentrations of transthyretin, a protein synthesized by choroid plexus, are nearly the same in ventricular and lumbar CSF [15].

There are no available reference values well established for Aβ42 ventricular levels in healthy individuals. Therefore, we measured Aβ42 levels in lumbar and ventricular CSF of 5 patients with chronic communicating hydrocephalus and we found that ventricular and lumbar Aβ42 values are not significantly different. Despite the small sample size, this fact suggests the ventricular CSF Aβ42 concentrations measured in this study seem to be relevant for a comparison with those existing in lumbar CSF of control subjects.

In iNPH, several studies [16-19] demonstrated that the lumbar Aβ42 levels are lowered. Our results are in good agreement with these previous data: more than 75% of the studied patients, whatever the aetiology of the hydrocephalus, exhibit reduced Aβ42 levels in their ventricular CSF. The fact that the ventricular Aβ42 levels were similar iNPH and oCH suggests some similarities in the pathophysiological process despite the existing differences in CSF dynamics and aetiologies. The same findings were reported for other proteins (neurofilament light protein, tau, sulfatide, vasoactive intestinal peptide and neuropeptide Y) in case of iNPH and aqueductal stenosis [20]. Several hypotheses could explain this decrease of Aβ42 levels.

Several hypothesis might be put forward to explain the decrease in Aβ42 levels. First, oligomer formation, favoured by the altered CSF turnover in NPH, could partially mask the antigenic sites of the peptide, especially the C-terminal part, which is hidden inside the hydrophobic core of the aggregate. This therefore could lead to an underestimation of the Aβ42 levels when using an ELISA technique [21]. Second, Aβ42 glycation could contribute to an underestimation of the protein concentration by ELISA. Indeed, the CSF glucose concentration is relatively high (about 0.5g/l), and CSF stasis promotes Amadori products formation [22], which could also mask some antigenic sites of the peptide. Third, a reduced production of Aβ42 could be suspected, as described in Creutzfeldt-Jacob's disease. In this affection, there are no Aβ42 deposits and CSF Aβ42 levels are significantly reduced [23], this probably due to the inhibition of β-secretase cleavage by PrP(C) [24].

However, an intra-cerebral sequestration of amyloid protein most likely occurs. Strozyk *et al*, investigating the relationship between amyloid neuropathology and post-mortem CSF Aβ42 levels in an autopsy sample of 155 patients with dementia found that a high number of plaques is associated with reduced levels of ventricular Aβ42 [25].

The « B compound of Pittsburgh » (PIB), a thioflavine derivative, which is a ligand of Aβ42, is able, after i.v. injection, to cross the blood brain barrier and bind to Aβ peptides. Molecular neuroimaging allows to detect amyloid pathology *in vivo *using PET scanning with PIB [26]. Several authors [27,28] using the PIB PET scanning, demonstrated, in patients with Alzheimer disease, that Aβ42 lumbar levels are lowered and inversely correlated to the Aβ load of brain. With the same technique, Leinonen *et al *[29] demonstrated the occurrence of amyloid deposits in 5/10 patients with iNPH, and cerebral biopsies performed during shunt surgery confirmed the existence of amyloid deposits in these patients. In a large follow-up study of NPH, idiopathic or secondary, Leinonen *et al *[30] also showed, on small cortical brain biopsies obtained during surgical treatment, that 186/433 (43%) patients displayed Aβ42 deposits. These lesions of AD are more frequent among older subjects with iNPH [31], which could be explained partially by a reduced transport *via *LRP-I [2,32] and lower activities of some enzymes involved in the catabolism of the Aβ42 peptide [33]; this suggests an increasing role of the CSF turnover in Aβ42 clearance during aging.

It is interesting to note, based on data derived from the ADNI database, an inverse correlation between ventricular size and CSF Aβ42 levels is observed in controls, MCI and AD, which also supports the hypothesis that the CSF turnover could play an important role in the regulation of the CSF Aβ42 peptide concentration [34].

## Conclusion

Our study shows that ventricular and lumbar levels of Aβ42 are not significantly different in surgical communicating hydrocephalus. Patients with iNPH exhibit a frequent decrease in ventricular CSF Aβ42 peptide levels, which is in good agreement with decreased lumbar levels previously described. This is consistent with an intra-cerebral sequestration of the peptide resulting from the reduced CSF turnover. Similarly, patients with surgical chronic hydrocephalus of other origins show an impairment of CSF turnover and also exhibit a decrease in Aβ42 ventricular levels. Thus, the decline in CSF Aβ42 concentration is not specific of iNPH and seems independent of the surgical hydrocephalus aetiology. This study suggests that the reduced CSF turnover is a key event in the decrease of Aβ42.

## Abbreviations

CSF: cerebrospinal fluid; iNPH: idiopathic normal pressure hydrocephalus; oCH: chronic hydrocephalus of other origins; PIB: B compound of Pittsburgh

## Competing interests

The authors declare that they have no competing interests.

## Authors' contributions

JMS conceived the study, led this project and drafted the manuscript with PJ and JCM. JP, AF, PT, DL, OG, and JMS selected patients. JP, AF, PT and DL removed CSF during surgery. NL, AMB, JCM performed assays. OG performed the statistical analysis. All authors read and approved the final manuscript. All the authors contributed equally to this work.
